# HIV-Related Stigma and Its Impact Across the HIV Care Continuum: Implications for Testing, Treatment, and Long-Term Retention

**DOI:** 10.7759/cureus.110535

**Published:** 2026-06-09

**Authors:** Samina Somji, Joshua Ngimbwa, Tevin Kahwa, Rukhsaar Ali, Caren Elias, Farah A Ebrahim, Reena Shah, Munish Sharma, Salim Surani

**Affiliations:** 1 Internal Medicine, Aga Khan University, Dar es Salaam, TZA; 2 Medicine, Aga Khan University, Nairobi, KEN; 3 Internal Medicine, Aga Khan University, Nairobi, KEN; 4 Pulmonary and Critical Care, Baylor Scott and White Medical Center - Temple, Temple, USA; 5 Medicine, Valley Coastal Bend Veterans Affairs (VA), Harlingen, USA; 6 Medicine, University of Houston, Houston, USA; 7 Anesthesiology, Mayo Clinic, Rochester, USA

**Keywords:** anticipated stigma, antiretroviral therapy, enacted stigma, hiv-related stigma, hiv testing, internalized stigma, retention in care, structural stigma, treatment adherence

## Abstract

While major medical breakthroughs have made human immunodeficiency virus (HIV) a manageable chronic illness, stigma related to HIV continues to create significant obstacles for prevention efforts, testing programs, treatment access, and ongoing patient care. This stigma affects people at personal, social, healthcare, and system-wide levels, preventing progress toward international HIV control objectives, particularly the Joint United Nations Programme on HIV/AIDS (UNAIDS) 95-95-95 targets, which aim for 95% of people living with HIV to know their HIV status, 95% of those diagnosed to receive sustained antiretroviral therapy (ART), and 95% of those receiving ART to achieve viral suppression.

The objective of this article is to examine different types, mechanisms, and consequences of HIV-related stigma and its impact across the HIV care continuum, including from initial testing to treatment initiation and sustained retention in care.

This comprehensive review examines current research on HIV-related stigma, drawing from theoretical models, population studies, and systematic analyses, with particular focus on sub-Saharan Africa (SSA) and Tanzania. Enacted, anticipated, internalized, and structural stigma were analyzed in relation to HIV testing participation, the initiation of antiretroviral therapy (ART), continued care engagement, and long-term health outcomes. Results showed that stigma surrounding HIV substantially decreases testing participation because people fear having their status revealed, facing discrimination, and being socially rejected. Following diagnosis, stigma leads to delays in starting ART, poor medication compliance, treatment gaps, and withdrawal from medical care. Self-directed stigma strongly correlates with depression and diminished confidence in managing one's health, while discrimination in healthcare environments and institutional barriers damage relationships between patients and providers. Vulnerable groups face multiple forms of stigma simultaneously, which worsens disparities in care participation and viral control. Research shows that comprehensive interventions targeting multiple levels effectively reduce stigma and enhance health outcomes.

HIV-related stigma continues to be a major public health challenge that undermines individual health outcomes, contributes to ongoing HIV transmission, increases healthcare utilization and costs, reduces workforce productivity, and impedes progress toward public health and economic goals. Tackling stigma through well-coordinated, multifaceted interventions is critical for increasing testing rates, encouraging treatment initiation, maintaining long-term patient engagement, and achieving lasting viral suppression to control the epidemic overall.

## Introduction and background

Over four decades, human immunodeficiency virus (HIV) has evolved from a universally deadly disease to a chronic condition that can be effectively managed, due to significant advances in medical research and public health approaches [1]. HIV primarily targets CD4-positive T lymphocytes, causing gradual immune system weakening and greater vulnerability to opportunistic infections when treatment is not provided [1]. The broad implementation of combination antiretroviral therapy (ART) has dramatically changed patient outcomes by enabling stable viral control, immune system recovery, and near-normal life expectancy [2].

Despite treatment and prevention improvements, stigma associated with HIV remains widespread globally. Data from the Joint United Nations Programme on HIV/AIDS (UNAIDS) shows that nearly one in five people with HIV experience discrimination in medical settings, while more than half report self-stigma in many countries with high HIV rates [3]. In sub-Saharan Africa (SSA), community surveys reveal that many adults still hold negative views toward people living with human immunodeficiency virus (PLHIV), including unwillingness to purchase food from HIV-positive vendors or permit children to attend school with HIV-positive peers [4]. Research in Tanzania specifically has documented substantial levels of both perceived and enacted stigma among PLHIV, with stigma closely linked to poor viral control and decreased life quality [5,6]. These results demonstrate that HIV-related stigma continues to be prevalent and undermines optimal HIV care despite access to effective ART [3].

Methodology

In this comprehensive review, we examined the prevailing literature related to HIV-related stigma. We drew inferences from theoretical models, population studies, and systematic analyses. We particularly emphasized the literature from the sub-Saharan region and Tanzania. Enacted, anticipated, internalized, and structural stigma were analyzed in relation to HIV testing participation, the initiation of antiretroviral therapy (ART), continued care engagement, and long-term health outcomes. A detailed Preferred Reporting Items for Systematic Reviews and Meta-Analyses (PRISMA) guideline was not followed as this is not a systematic review.

## Review

Tanzania's implementation of the "test and treat" approach has enhanced early HIV detection and prompt ART initiation [5]. Current treatment regimens, particularly those dolutegravir-based therapies, are highly effective, well-tolerated, and associated with better compliance [2]. However, nonmedical obstacles, including HIV-related stigma, the fear of status disclosure, and social discrimination, continue to prevent testing participation, treatment initiation, and sustained care engagement. Addressing these social factors remains essential for HIV epidemic control in an era of effective treatment [5].

HIV-related stigma continues to pose a major challenge to effective HIV prevention, treatment, and ongoing care worldwide. It describes the social devaluation and discrimination that PLHIV face due to their HIV status [7]. Various forms of stigma consistently correlate with reduced HIV testing participation, delayed connection to care, poor ART adherence, and loss from follow-up care, creating a significant barrier to reaching the UNAIDS 95-95-95 targets, which specify that 95% of all PLHIV should know their status, 95% of diagnosed individuals should receive sustained ART, and 95% of those on ART should achieve viral suppression [3].

Sub-Saharan Africa carries a disproportionate HIV and stigma burden, with moral judgments and fears driving negative attitudes [3,4]. Even with the major expansion of HIV services in Tanzania, stigma continues and correlates with delayed care and inadequate viral suppression, emphasizing the need to address social factors, alongside medical interventions [6].

Achieving better individual outcomes and population-level HIV epidemic control requires access to HIV testing combined with ART initiation and continuous long-term care [8]. Early testing for timely diagnosis and ART initiation before severe immune suppression develops is necessary to reduce HIV-related opportunistic infections, illness, and death. People who begin treatment early are more likely to achieve consistent viral suppression, maintain immune function, and have a good life quality. In contrast, those with delayed testing, often caused by HIV-related stigma and discrimination fears, frequently present late and experience worse clinical outcomes [1].

Broader testing access and effective ART support HIV prevention at the public health level. Sustained viral suppression eliminates sexual transmission risk, known as "Undetectable = Untransmittable (U=U)," and forms the foundation of global HIV control strategies promoted by the World Health Organization (WHO) and UNAIDS [8]. Long-term engagement in care remains vital because HIV requires lifelong treatment, support in adherence, and continuous clinical monitoring. For achieving a sustained advantage at both the individual and population level, addressing the social and structural barriers to testing, treatment, and retention in care is crucial.

Substantial barriers to care continue among PLHIV despite HIV being known as a chronic and manageable condition due to advances in diagnostic testing and ART [8]. HIV-related stigma remains an important factor influencing individuals' willingness to seek HIV testing, initiate treatment, and remain engaged in long-term HIV care. HIV-related stigma causes individuals not to access HIV testing because they fear discrimination and social exclusion, thus leading to delays in initiating treatment after the diagnosis is made, and it also hinders them from seeking continued care [4]. These interconnected psychological, social, and structural barriers hinder the full benefits of effective HIV services, including in settings where testing and treatment are commonly available. As a result, HIV-related stigma significantly causes delays in access to early diagnosis, the initiation of treatment, and continued care, causing a major challenge to achieving global HIV control targets and ending the HIV epidemic [3].

Understanding HIV-related stigma

HIV-related stigma refers to negative beliefs, attitudes, and discriminatory actions directed toward individuals living with or associated with HIV [9]. Despite medical advances that have transformed HIV into a manageable chronic condition, stigma remains deeply rooted within social, cultural, and institutional systems. Understanding its origins, forms, and intersections is critical to explaining how it challenges access to testing, treatment, and sustained care [9].

The roots of HIV-related stigma can be traced to the early years of the epidemic in the 1980s; it was then that HIV was first identified among marginalized populations, specifically in gay men, sex workers, people who inject drugs, and, then later, certain racial and ethnic minorities [10]. Transmission was closely linked to sexuality and drug use behaviors heavily moralized in many societies, and hence, the disease quickly became linked to deviance and immorality. Media portrayals frequently emphasized fear and blame, framing HIV as a consequence of "risky" or "sinful" behavior rather than a public health issue. These premature narratives are shaped by enduring stereotypes and moral judgments that continue to influence public attitudes even today [10,11].

Consequently, HIV stigma developed not merely from the fear of infection but as a socially constructed phenomenon rooted in power, inequality, and moral regulation [11]. This historical context explains why stigma continues to exist even in the era of effective ART.

HIV-related stigma manifests in several interrelated forms. Enacted stigma refers to overt discrimination, exclusion, or mistreatment directed at PLHIV [9]. It includes healthcare service denial, violations of confidentiality, social rejection, employment discrimination, and violence. In healthcare settings, enacted stigma may manifest as judgmental attitudes, delay in services, or the unnecessary use of protective measures. Such experiences hinder individuals from seeking testing or continuing with care [12].

Anticipated stigma involves the expectation of bias or negative reactions when one's HIV status is known. Even in the absence of direct discriminatory events, the fear of social rejection, relationship loss, or community exclusion may prevent individuals from revealing their status, attending clinics, or even collecting their medications regularly. Anticipated stigma constitutes a powerful psychological hurdle that directly affects testing and continuation in care [9].

Internalized stigma is when individuals living with HIV absorb societal negative beliefs and direct them toward themselves. It can manifest as guilt, shame, decreased self-worth, depression, or social withdrawal. Internalized stigma has been coupled with inadequate ART adherence, delayed care-seeking, and mental health disorders, undermining one's self-efficacy and resilience [9].

Structural stigma refers to societal-level conditions, cultural norms, laws, and institutional policies that constrain opportunities and resources for PLHIV. This may manifest as the criminalization of HIV transmission, penalizing laws against gay relationships or sex workers, the absence of anti-discrimination protections, and healthcare policies that compromise confidentiality. Structural stigma reinforces social inequality and creates systemic obstacles to testing, treatment initiation, and sustained treatment [13]. These forms of stigma frequently interact and reinforce one another, creating multilayered barriers across individual, interpersonal, and institutional levels [13].

HIV-related stigma rarely operates in isolation. It overlaps with other stigmatized identities such as race, gender, sexual character, socioeconomic status, and substance use. An example includes women living with HIV who may experience compounded stigma linked to gender norms and expectations regarding sexual morality [14]. Gays may face dual stigma related not only to their HIV status but also to their sexual orientation. Poverty further amplifies vulnerability in resource-limited settings, resulting in limited access to supportive services. An intersectionality framework highlights how multiple systems of discrimination interact to produce heightened vulnerability. Individuals belonging to multiple marginalized groups often experience more severe barriers to testing and continued care, thus contributing to disparities in HIV outcomes (Table 1) [14].

**Table 1:** The main forms of HIV stigma and how they affect testing uptake, treatment initiation, and long-term care retention. HIV, human immunodeficiency virus; ART, antiretroviral therapy

Type of Stigma	Definition	Impact on HIV Testing	Impact on Treatment Initiation	Impact on Retention and Adherence
Enacted stigma	Direct discrimination, exclusion, or mistreatment due to HIV status	Avoidance of testing due to the fear of discrimination or breach of confidentiality	Delayed initiation of ART due to negative experiences in healthcare	Avoidance of clinics, missing appointments, or complete disengagement from care
Anticipated stigma	Expectation of discrimination or rejection if HIV status becomes known	Reluctance to seek testing or avoidance resulting in delays in testing due to the fear of disclosure	Reluctance to initiate ART	Treatment interruption due to skipping visits or refills of ART due to the fear of being identified
Internalized stigma	Negative societal beliefs about HIV, leading to self-blame and shame	Poor motivation to seek testing	Psychological distress affecting the initiation of ART	Treatment interruption and poor ART adherence
Structural stigma	Institutional laws, policies, and social norms that restrict opportunities or rights	Reduced stigma-free testing services and confidentiality and hence reduced uptake in testing	Delayed initiation of treatment due to criminalization, discrimination, or restrictive policies	Long-term disengagement in care due to systemic barriers and the lack of protection

Impact of stigma on access to HIV testing

HIV testing represents a critical first step in the HIV care continuum. Without diagnosis, people cannot access ART, reach viral suppression, or gain from treatment's preventive benefits. HIV-related stigma significantly reduces testing uptake across social, psychological, and structural [15].

The fear of being seen publicly creates an immediate barrier to HIV testing. In many locations, testing centers are recognizable facilities, so being observed entering or leaving these places may raise questions about someone's HIV status. People worry about rumors, confidentiality violations, or damage to their reputation [7].

This concern becomes especially pronounced in close-knit communities, rural areas, and places where HIV carries heavy stigma. Even when testing services exist, privacy concerns prevent people from seeking them. Research consistently shows that perceived stigma correlates with lower rates of voluntary HIV testing [3,12].

Anticipated stigma strongly influences delays in HIV testing. People fear rejection from family, partners, employers, or healthcare workers. Testing becomes viewed not as a medical procedure but as a potential threat to identity, relationships, and employment. This fear can lead people to avoid testing entirely, postpone it repeatedly despite knowing their risk, or seek services at anonymous or distant locations [14].

Research shows that higher anticipated stigma connects to lower testing rates and delayed diagnosis, especially among key populations such as gay men, sex workers, and people who inject drugs [7,14].

Stigma also undermines public health efforts to make HIV testing routine, including provider-initiated testing, community outreach, and home-based programs. Even when testing is regularly offered in prenatal clinics or outpatient settings, many people refuse due to the fear of being labeled HIV-positive [3]. Structural stigma and past negative healthcare encounters further reduce trust in health systems. Communities with high stigma levels show lower uptake of routine HIV testing services and make slower progress toward global targets such as the UNAIDS 95-95-95 goals [3].

Delayed or avoided testing creates serious clinical and public health problems. People diagnosed late are more likely to present with advanced immune suppression, low CD4 counts, opportunistic infections, and AIDS-defining conditions. Late diagnosis is linked to increased hospitalizations, higher illness rates, and earlier death even after starting ART [15]. Individuals unaware of their HIV status may unknowingly transmit HIV to sexual partners. Early HIV diagnosis and immediate ART initiation reduce viral load to undetectable levels, effectively lowering sexual transmission risk (U=U principle). Therefore, stigma-related testing delays create undiagnosed HIV infection sources and hamper epidemic control efforts [15].

Impact of stigma on access to HIV treatment

After the initial HIV diagnosis, rapid care linkage and early ART initiation are essential for achieving viral suppression and reducing transmission. Stigma continues to have strong effects even after people learn their HIV status. Newly diagnosed individuals may delay starting ART, avoid returning to clinics for follow-up and medication refills, seek care far from their communities, or avoid care services entirely [16]. The fear of disclosure, anticipated discrimination, and internalized shame can make lifelong treatment feel psychologically overwhelming. Perceived and internalized stigma strongly associate with delayed ART initiation and poor care linkage [16].

Enacted stigma in healthcare settings greatly shapes future care-seeking behavior. People who face discrimination, such as disrespect, neglect, or moral judgment, are less likely to return for follow-up visits. For marginalized populations, compounded stigma may lead to the complete avoidance of formal healthcare services. Healthcare-related stigma is linked to reduced healthcare facility retention and lower ART adherence [17].

Judgmental attitudes further damage trust and limit open communication, including nonverbal signals that may reinforce stigma and weaken therapeutic relationships. Confidentiality breaches intensify disengagement. Patients worry about a lack of privacy during consultations, the obvious labeling of HIV services, or unauthorized status disclosure, which amplifies anticipated stigma [3].

Stigma dramatically affects ART adherence through multiple pathways: patients may hide medications, skip ART doses around others, delay pharmacy refills, or experience depression and reduced self-care motivation due to internalized stigma. Meta-analytic evidence shows consistent connections between HIV-related stigma and poor ART adherence [18].

Mental health serves as a crucial pathway between stigma and treatment outcomes. Internalized stigma strongly correlates with anxiety, depression, substance abuse, and social withdrawal. Depression is linked to poor ART adherence and lower viral suppression, creating a reinforcing cycle where stigma worsens mental health, leading to poorer adherence and potentially more visible illness, which further increases stigma [19].

Impact of stigma on long-term HIV care and retention

HIV-related stigma remains a major factor in poor continued HIV care engagement, with effects extending beyond diagnosis and ART initiation. Sustained HIV care requires regular clinic visits, good ART adherence, and social network support, but stigma disrupts all these processes. Studies have reported that PLHIV experiencing moderate to high stigma have approximately 1.5-fold to twofold greater odds of delayed ART initiation, treatment interruption, and loss to follow-up compared with those reporting lower levels of stigma [18,19]. Enacted stigma from family members, partners, employers, and communities often causes social isolation and withdrawal of emotional and material support, further reducing retention in care [14].

Internalized stigma affects mental health, self-efficacy, and motivation and is strongly linked to poor adherence and long-term care withdrawal, regardless of clinical status [14,20]. Structural stigma, including criminalization and discriminatory workplace practices, discourages healthcare-seeking and disclosure, particularly in resource-limited settings [20]. Consequently, stigma-related care disruptions lead to poor viral suppression and negative clinical outcomes, highlighting stigma as a critical factor in HIV treatment success [6].

Coping mechanisms and protective factors

Coping mechanisms and protective factors are crucial for reducing negative effects related to HIV stigma and in supporting sustained care among PLHIV. Peer support and community-based organizations improve psychological well-being, treatment adherence, and care continuation by providing shared experiences, emotional validation, and practical guidance for managing daily HIV-related challenges [18,21]. Peer-led interventions foster belonging and help normalize living with HIV, reducing internalized stigma and social isolation [21].

Supportive healthcare environments serve as equally important protective factors. Health facilities practicing confidentiality, nondiscrimination, and respectful patient-provider interactions build healthcare system confidence and encourage timely HIV testing, treatment initiation, and regular follow-ups [17]. Good provider attitudes and communication have been shown to be linked to influencing patient disclosure, good ART adherence, and the continuity of care, especially in stigmatized populations [17].

Education is critical in building resilience to HIV stigma. Knowledge about HIV transmission, effective treatment, and the concept of viral suppression reduces fear, removes any misinformation, and enables PLHIV to challenge stigmatizing beliefs [9]. Empowerment-based interventions are essential for strengthening self-efficacy, promoting health-seeking behaviors, and supporting informed decision-making regarding HIV disclosure and engagement in care [9].

Another important coping mechanism is the use of disclosure strategies. When disclosure is selective, planned, and directed toward trusted individuals, it can provide emotional support, improve adherence to antiretroviral therapy (ART), and reduce psychological distress. However, when disclosure is unplanned or coerced, it may exacerbate stigma and negatively impact social relationships [22].

Strong social support systems with family, partners, peers, and community networks reduce the impact of stigma and often result in better mental health outcomes and sustained continuation in care [18,22]. All these outlined protective factors reiterate the importance of integrating psychosocial support together with medical interventions in comprehensive HIV care [22].

Interventions to reduce stigma and improve access

Reducing HIV-related stigma and improving access to testing, treatment, and long-term care require a well-coordinated intervention at individual, healthcare, and structural levels. Education aimed at reducing stigma and public awareness campaigns play an essential role in addressing misinformation, fear, and moral judgments associated with HIV. Evidence has shown that population-level educational interventions that emphasize the effectiveness of treatment and the concept of viral suppression improve HIV-related knowledge, normalize HIV testing, and help reduce discriminatory attitudes [3,23].

Training healthcare workers to provide stigma-free, patient-centered care is critical for improving healthcare experiences and outcomes for people living with HIV (PLHIV). Interventions focusing on confidentiality, respectful communication, and nondiscriminatory clinical practices have been shown to reduce enacted stigma within healthcare facilities, strengthen the patient's trust in the provider, and improve the continuation of care [17]. In high-burden settings, healthcare worker training is especially resourceful because there is the fear of mistreatment, which remains a major hindrance to the utilization of services [17].

Policy and legal reforms play an important role in addressing structural stigma. Laws that criminalize HIV transmission, limit disclosure, or disregard key populations reinforce fear and discourage patients with HIV from engagement with health services. Examples include laws criminalizing HIV exposure or transmission, mandatory HIV disclosure requirements, and legislation that penalizes same-sex relationships, sex work, or drug use, all of which may discourage engagement with HIV services [24].

The provision of HIV services in the general healthcare settings further helps to reduce the burden of stigma by considering HIV as any other chronic illness. The integration of HIV services into primary care clinics, maternal and child health services, tuberculosis clinics, sexual health services, and chronic disease management programs can reduce stigma and improve the continuity of care [25]. Finally, community-led and rights-based approaches empower affected populations to actively participate in the design, implementation, and evaluation of HIV programs, thereby enhancing cultural appropriateness, community trust, and the long-term sustainability of interventions. Community ownership has been shown to reinforce trust, increase service uptake, and reinforce dignity and autonomy among PLHIV, which makes it a foundation of effective stigma reduction strategies [3,24].

## Conclusions

HIV-related stigma continues to exert a major influence across the HIV care continuum, negatively affecting the uptake of testing, timely ART initiation, and continuous long-term engagement in care. Despite major medical advances that have transformed HIV into a chronic and manageable condition, stigma continues to remain a persistent barrier that challenges the full benefits of effective testing and treatment services. The fear of discrimination, social exclusion, and unwanted disclosure discourages health-seeking behavior, contributes to treatment interruptions, and compromises continued retention in care, ultimately affecting viral suppression and the quality of life of PLHIV.

Failure to address HIV-related stigma has significant public health implications, including ongoing HIV transmission, the increased risk of drug resistance, and missed opportunities to achieve global HIV control targets. These challenges highlight that medical interventions alone are insufficient to end the HIV epidemic. Comprehensive, multi-level strategies are required, including anti-stigma education, stigma-free healthcare delivery, legal and policy reforms, and community-led, rights-based approaches. Addressing stigma as a structural and social determinant of health is essential to improving access to care, strengthening long-term health outcomes, and advancing equitable and sustainable HIV responses.
